# New-Onset Diabetes Mellitus after Kidney Transplantation

**DOI:** 10.3390/jcm13071928

**Published:** 2024-03-27

**Authors:** Salah Alajous, Pooja Budhiraja

**Affiliations:** Division of Medicine, Mayo Clinic Arizona, Phoenix, AZ 85054, USA; alajous.salah@mayo.edu

**Keywords:** NODAT, transplant, kidney, HbA1c, GLP1 AR, metformin

## Abstract

New-Onset Diabetes Mellitus after Transplantation (NODAT) emerges as a prevalent complication post-kidney transplantation, with its incidence influenced by variations in NODAT definitions and follow-up periods. The condition’s pathophysiology is marked by impaired insulin sensitivity and β-cell dysfunction. Significant risk factors encompass age, gender, obesity, and genetics, among others, with the use of post-transplant immunosuppressants intensifying the condition. NODAT’s significant impact on patient survival and graft durability underscores the need for its prevention, early detection, and treatment. This review addresses the complexities of managing NODAT, including the challenges posed by various immunosuppressive regimens crucial for transplant success yet harmful to glucose metabolism. It discusses management strategies involving adjustments in immunosuppressive protocols, lifestyle modifications, and pharmacological interventions to minimize diabetes risk while maintaining transplant longevity. The importance of early detection and proactive, personalized intervention strategies to modify NODAT’s trajectory is also emphasized, advocating for a shift towards more anticipatory post-transplant care.

## 1. Introduction

Kidney transplantation is associated with a better quality of life and overall survival than being on dialysis [1]. However, it is crucial to recognize that this life-improving procedure comes with its set of metabolic challenges, most notably the risk of NODAT [2]. NODAT is a common complication after transplants associated with adverse effects on the patient and graft survival.

The development of NODAT is intricately linked to the use of essential immunosuppressive medications post-transplantation, such as glucocorticoids and calcineurin inhibitors (CNIs) [3]. Moreover, the risk factors for NODAT extend beyond the pharmacological implications of post-transplant care. They also include elements common to the general population predisposing individuals to type 2 diabetes, such as age, family history of diabetes, ethnicity, and pre-transplantation glucose intolerance. Additionally, transplant-specific factors that exacerbate these risks [4] include the type of organ transplanted, the intensity of the immunosuppressive regimen required, and the overall health and pre-existing conditions of the transplant recipient. NODAT not only affects quality of life but is also associated with increased patient and graft loss. Understanding the multifaceted nature of NODAT’s etiology is crucial for the medical community. This requires the careful selection and management of immunosuppressive therapies, regular monitoring of blood glucose levels, and lifestyle interventions that can help reduce the overall risk of diabetes. Through such comprehensive care strategies, healthcare providers can significantly contribute to improving both the quality and longevity of life for kidney transplant recipients. 

Our review article aims to delve into the intricate realm of NODAT in kidney patients, unraveling its complex pathophysiology while shedding light on diverse treatment modalities. 

## 2. Prevalence, Diagnosis, and Risk Factors

### 2.1. Prevalence and Diagnostic Criteria for NODAT in Kidney Transplant Recipients

Following a kidney transplant (KT), around 10% to 40% of patients experience NODAT, depending on the year after the transplant and the method for diagnosis [5,6]. The incidence of NODAT was approximately 10% to 20% at 1 year and 25% to 35% at 3 years after KT [7]. The incidence of NODAT is higher in non-kidney organ transplant recipients. The incidence of NODAT in heart transplant recipients ranges between 20% and 30%; in liver transplant recipients, 20–40%; and in lung transplant recipients, 20–40% in the first 5 years [8]. 

The first clear diagnostic criteria for NODAT were introduced in 2003 by the American Diabetes Association and the World Health Organization [9]. This includes a fasting glucose ≥ 126 mg/dL (7 mmol/L) on more than one occasion, random glucose ≥ 200 mg/dL (11.1 mmol/L) with symptoms, or a 2 h glucose level after a 75 g oral glucose tolerance test (OGTT) of ≥200 mg/dL (11.1 mmol/L) or HbA1C > 6.5% (48 mmol/mol) (Table 1). NODAT may be underdiagnosed amid significant anemia rates and a reliance on Hba1c [10].

### 2.2. Risk Factors for NODAT

NODAT development involves multiple factors, primarily centered around β-cell dysfunction alongside insulin resistance. The use of immunosuppressive drugs with diabetogenic effects can contribute to NODAT, particularly in high-risk patients [11]. Table 2 outlines the risk factors for NODAT, which can be divided into two groups—modifiable and non-modifiable. Non-modifiable risk factors include age, ethnicity, male sex, and family history of diabetes, whereas modifiable risk factors include immunosuppression, rejection episodes, obesity, and hepatitis C virus infection [12].

#### 2.2.1. Age

Advanced age and an extended duration of dialysis have been identified as factors contributing to an elevated risk of developing NODAT. These factors are closely associated with a decline in the Psoas Muscle Index (PMI) and an increase in intermuscular adipose tissue, both indicators of deteriorating muscle health and quality. Research indicates that a lower PMI correlates with higher levels of high-molecular-weight adiponectin, which can exacerbate insulin resistance and elevate the risk of NODAT [13]. 

In addition to the loss of muscle mass, aging is linked to a reduction in the efficiency of glucose transport into muscle cells, primarily due to the decreased functionality of the GLUT4 transporter. This impairment is compounded by increased adiposity and inflammation commonly associated with aging, further contributing to insulin resistance in elderly individuals [14]. Moreover, increased age in transplant recipients is also tied to enhanced apoptosis in aging islet β-cells. This cellular decline results in diminished insulin secretion, posing a higher risk of NODAT, particularly in recipients aged 45 years or older [15]. Understanding these interconnected factors is crucial for managing and mitigating the risk of NODAT in the transplant population, especially among older recipients.

#### 2.2.2. Sex

Males are at a heightened risk of developing NODAT compared to their female counterparts. This increased susceptibility in males can be attributed to a combination of genetic, hormonal, and physiological factors [16,17]. Males typically have a higher prevalence of visceral obesity—fat stored within the abdominal cavity—which is a known risk factor for insulin resistance and type 2 diabetes [18,19]. Conversely, females are found to have a risk of NODAT due to lower muscle mass, resulting in reduced blood sugar uptake [13].

#### 2.2.3. Genetic Predisposition to NODAT

Over the past two decades, studies have highlighted the increased risk of type 2 diabetes associated with genetic predisposition, although the risk tied to each gene is relatively small [20]. Several studies have identified an association between NODAT in kidney transplant recipients and specific common single nucleotide polymorphisms (SNPs). These SNPs are found in genes responsible for proteins involved in β-cell apoptosis, ATP-sensitive potassium channels, adiponectin, leptin, inflammatory pathways, and elements of the innate immune system. Individuals carrying multiple SNPs in diabetic genes faced an amplified risk. NODAT has also been correlated with SNPs in various IL genes, notably IL-2, IL-7R, IL-17R, IL-1B, IL-4, IL-17-RE, IL-17R, and IL-17RB [21]. Additionally, SNPs in transcription factor NFATc4 and adiponectin were associated with NODAT [22,23]. Limited literature suggests that African Americans and Hispanics face a higher susceptibility to developing NODAT in comparison to Caucasians. The risk ratio for NODAT is elevated by 32–68% in African American patients and 35% in Hispanic patients when compared to Caucasian patients [16].

#### 2.2.4. Obesity

NODAT exhibits a strong connection with visceral obesity, leading to the generation of inflammatory cytokines such as interleukin-6 and tumor necrosis factor-α, along with reduced levels of adiponectin associated with insulin resistance [19,24,25]. Visceral fat demonstrates resistance to the lipolytic effects of insulin and, upon mobilization, can impede liver metabolism. This interference may result in heightened hepatic gluconeogenesis, diminished apolipoprotein B degradation, and increased production of triacylglycerol-rich lipoproteins [26].

#### 2.2.5. Immunosuppressive Agents

Various immunosuppressive agents have well-documented diabetogenic effects of corticosteroids, calcineurin inhibitors, and mammalian target of rapamycin (mTOR) inhibitors. These are detailed below.

#### 2.2.6. Viral Infection

Hepatitis C virus (HCV) infection poses a risk for NODAT [27], as observed in a retrospective study involving 427 kidney transplantation recipients [28]. The heightened risk of NODAT associated with HCV infection may be attributed to multiple factors, such as the direct replication of the HCV virus in pancreatic islet β-cells leading to dysfunction and autoimmune destruction, causing inadequate insulin synthesis and secretion; direct damage to liver cells by HCV, resulting in impaired insulin utilization and insulin resistance; and the potential impact of HCV on the synthesis of insulin-related proteins through the mediation of the insulin signaling pathway, ultimately affecting insulin secretion [29].

## 3. Pathophysiology of NODAT

**Figure 1:**
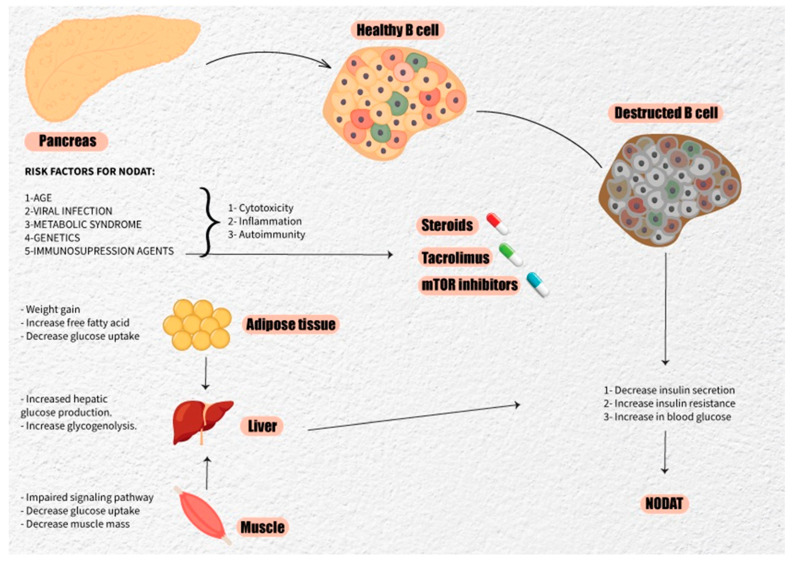
The figure illustrates the risk factors and the main pathophysiological mechanisms implicated in the pathogenesis of NODAT.

NODAT exhibits similarities to pre-transplant diabetes mellitus but encompasses other distinct mechanisms leading to hyperglycemia, marking it as a separate entity due to post-transplant risk factors that accelerate the risk of developing diabetes. Impaired insulin sensitivity [30,31], abnormal insulin production from pancreatic β-cell dysfunction [32,33], uncontrolled glucagon release [34], hypertriglyceridemia, and obesity all contribute to the risk for NODAT. Although the dominant pathophysiological process behind NODAT is debated, some groups highlight β-cell dysfunction as a primary mechanism [32,33] (Figure 1).

### 3.1. Insulin Resistance

Factors contributing to reduced insulin sensitivity in transplant recipients include age, male sex, obesity, and renal function [35]. Medications commonly used in transplantation, such as CNIs and rapamycin, have been associated with heightened insulin resistance. Cyclosporine A and tacrolimus, at therapeutic levels, hinder glucose uptake independently of insulin signaling by accelerating endocytosis, thereby leading to GLUT4’s removal from cell surfaces, and, thus, fostering insulin resistance [36]. The calcineurin/NFAT pathway, among others, controls gene transcription in skeletal muscle, with its inhibition potentially promoting insulin-resistant myosin fast fibers and contributing to insulin resistance onset [37].

Insulin resistance and insulin deficiency work together to cause hyperglycemia after kidney transplantation [30]. Studies tracking insulin sensitivity post-transplantation reveal a decline early on, showing defects in insulin secretion that correlate with glucose tolerance and the development of NODAT. Patients with NODAT sustain reduced insulin secretion even years after transplantation, while differences in insulin sensitivity become less pronounced than those with normal glucose levels. This sustained decrease in insulin secretion might stem from the ongoing use of immunosuppressive medications that harm β-cells, while insulin sensitivity tends to fluctuate. 

### 3.2. β-Cell Dysfunction

β-cell dysfunction plays a major role in the pathogenesis of NODAT [32]. The development of NODAT is closely linked to β-cell dysfunction, which is influenced by factors such as glucolipotoxicity, reduced insulin biosynthesis and secretion, and decreased β-cell mass. This dysfunction is often a result of β-cell dedifferentiation, a process wherein cells regress from a specialized to a less specialized state under stress, involving critical transcription factors [38,39]. This dedifferentiation serves as a defensive mechanism against various stresses, including glucolipotoxicity, oxidative and endoplasmic reticulum stress, mitochondrial dysfunction, and inflammation. During this process, cells revert to a more primitive developmental stage without undergoing cell death, thereby altering their form and function [40].

Before transplantation, B-cell damage may already be present and play a role in NODAT, as demonstrated by research. Immunosuppressive medications, notably tacrolimus and cyclosporine A, contribute to NODAT, particularly in patients with pre-transplant risk factors such as obesity and dyslipidemia. These drugs hinder insulin secretion and content, especially in individuals with pre-existing damage to β-cells, underscoring the significance of prior dysfunction in the development of NODAT [40]. Proinsulin, a marker indicating pancreatic β-cell dysfunction, reflects β-cell stress when insulin demands surpass current capacity, preceding a diabetes diagnosis and serving as a predictor for NODAT development, even with normal glucose levels. Elevated proinsulin levels (>10 pmpl/L) signify insulin resistance, as demonstrated by the IRIS-II study [41], with 80% of renal transplant recipients in the pancreatic β-cell dysfunction risk for NODAT study surpassing this threshold, suggesting a compromised β-cell function due to metabolic demands and prolonged exposure to immunosuppressive medications, thereby heightening NODAT risk for transplant recipients [33].

Insulin resistance and β-cell dysfunction are early indicators in the progression of diabetes, characterized by a hyperbolic relationship influenced by a negative feedback loop. Normally, pancreatic β-cells adjust to variations in insulin sensitivity, maintaining near-normal plasma glucose levels through compensatory increases in insulin secretion in healthy individuals. However, kidney transplant recipients exhibit heightened insulin resistance, influenced by factors like obesity, waist-to-hip ratio, and prednisolone treatment, as demonstrated by Oterdoom et al. [35].

### 3.3. Role of Immunosuppressive Medications

Immunosuppressive medications such as glucocorticoids, CNIs, and the mammalian target of rapamycin inhibitors significantly contribute to the onset of NODAT [7,42,43,44] and hold greater significance than conventional risk factors.

#### 3.3.1. Glucocorticoids

The process behind how glucocorticoids cause high blood sugar involves several factors: they make the body more resistant to insulin, decrease insulin production, and increase the creation of new glucose. They trigger the liver to generate more glucose by activating specific genes and boosting the impact of hormones like glucagon and epinephrine [45]. Glucocorticoids also disrupt normal appetite control, resulting in weight gain and further insulin resistance. The question of whether steroids induce weight gain or if it stems from improved appetite due to reduced uremia remains a topic of debate. 

Although all forms of glucocorticoids can raise blood sugar levels, it is more likely to happen with higher doses and longer treatment periods [45]. The administration of high-dose glucocorticoids, specifically at a dosage of 10 mg or higher, has the potential to trigger insulin resistance. Information gathered from registries suggests that regimens devoid of steroids carry a reduced risk of developing DM [46]. In a study encompassing 57 KTRs, the gradual reduction of prednisolone dosages from 10 mg to 5 mg daily resulted in improved insulin sensitivity. Notably, completely discontinuing prednisolone did not yield any additional enhancement in insulin sensitivity [47].

#### 3.3.2. CNIs

CNIs, a set of medications used to suppress the immune system in autoimmune diseases and post-organ transplants, play a vital role in preventing T-cell-mediated immune reactions. However, while their primary function is the inhibition of immune responses, these drugs can lead to elevated blood sugar levels due to their interference with calcineurin phosphatase activity, inhibiting insulin production by β-cells. This enzyme is not only present in immune cells but is also distributed in tissues like the pancreas, impacting insulin secretion and resulting in hyperglycemia. The process leading to high blood sugar involves multiple stages of insulin secretion, from mRNA transcription to post-translational modification [48].

The effect of CNIs on blood sugar varies significantly among individuals. The degree of hyperglycemia is influenced by the dosage and the specific medication used. Tacrolimus has been linked to higher rates of elevated blood sugar compared to cyclosporine. This difference might be due to the differing concentrations of their binding proteins in various tissues. Tacrolimus-binding protein is highly concentrated in pancreatic cells, affecting insulin production, while cyclosporine-binding protein is more prevalent in other organs like the heart, liver, and kidneys [49,50].

#### 3.3.3. Sirolimus

mTOR inhibitors diminish insulin signaling by suppressing the phosphorylation of protein kinase B in liver, fat, and muscle cells and induce β-cell apoptosis [51]. The administration of Sirolimus, an inhibitor targeting the mammalian target of rapamycin (mTOR), has been recognized as a contributing factor in the emergence of NODAT. Johnston et al. conducted an analysis using USRDS data, revealing that Sirolimus, irrespective of its combination with a calcineurin inhibitor or an antimetabolite like mycophenolate mofetil or azathioprine, was independently linked to an elevated risk of NODAT [52]. Meanwhile, Teutonico et al. observed in a prospective study involving renal transplant recipients that substituting calcineurin inhibition with Sirolimus exacerbated insulin resistance and diminished insulin responsiveness. Notably, these disruptions in glycemic control were correlated with an escalation in serum triglyceride levels, a well-known side effect associated with the use of mTOR inhibitors [53].

#### 3.3.4. Everolimus

A recent meta-analysis examining the combination of CNIs plus mTOR inhibitors in new KTRs found no significant increase in the incidence of NODAT at the 1-year mark compared to CNIs plus antiproliferative agents across 13 studies involving 4561 participants (relative risk 1.16, 95% confidence interval 0.97–1.38, *p* = 0.10) [54]. These findings were corroborated by the TRANSFORM study, a 24-month prospective trial involving 2037 new KTRs randomized to receive Everolimus with reduced-exposure CNI versus mycophenolate with standard-exposure CNIs, where no difference in NODAT incidence was observed (risk ratio 1.09, 95% CI 0.87–1.37), along with comparable efficacy and graft function [55]. Despite the absence of variance in NODAT incidence, the potential cause could be attributed to the reduction in CNI dosage.

## 4. Management and Treatment

### 4.1. Pharmacological Management of NODAT

#### 4.1.1. Immunosuppressive Drug Adjustments

The initiation of an immunosuppressive regimen before a kidney transplant aims to lower the recipient’s immune function. The tailored selection of this regimen hinges on the recipient’s circumstances and the perceived immunological risk. Presently, there is no consensus on the preferred immunosuppression regimen to prevent NODAT. However, in the modern era of immunosuppression, the regimen should balance immunologic risk against potential complications like NODAT in kidney transplant recipients [43,56,57]. In the HARMONY trial, which centered on recipients treated with tacrolimus and mycophenolic acid, it was discovered that the swift withdrawal of corticosteroids decreased the occurrence of NODAT without elevating the risk of rejection [58]. 

Younger and overweight patients experience advantages from steroid avoidance protocols, particularly when the risk of rejection is offset by T-cell-depleting therapy (such as TMG/ALEM), as highlighted in a study published in Kidney Med [46]. Moreover, the response to steroid withdrawal appears to differ based on age. In older Solid Organ Transplant recipients, a population-based study indicated more favorable outcomes, including reduced NODAT and mortality, following early steroid withdrawal. However, this benefit is tempered by an elevated risk of rejection. A registry analysis involving 6070 KTRs emphasized that discontinuing steroids after anti-IL-2 induction therapy within the initial 18 months post-transplantation correlated with a heightened risk of graft loss compared to continuous steroid maintenance. Considering these findings, the decision to pursue a steroid avoidance regimen should be carefully assessed against potential graft-related risks. When contemplating steroid avoidance, induction therapy with lymphocyte depletion may represent a prudent approach [59].

Immunosuppressive drugs significantly contribute to NODAT development. Customizing these drugs could mitigate the risk of NODAT, but such adjustments should not jeopardize graft survival. Decreasing corticosteroid dosage during the initial post-transplant year has notably enhanced glucose tolerance. The swift tapering of steroids in patients with low immunological risk, a high NODAT risk, and personalized steroid-sparing protocols show promise [60]. Retrospective trials favoring corticosteroid-sparing regimens at discharge exhibited significant benefits concerning NODAT at three years compared to corticosteroid-containing regimes [61]. For patients at high NODAT risk but low immunological risk, the other choices of immunosuppressive regimen include belatacept-based or cyclosporine therapies. In cases where it is difficult to control NODAT, a switch from tacrolimus to cyclosporine might be beneficial [9]. Moreover, the effective management of renal transplant patients involves baseline assessments pre-transplantation and personalized immunosuppressive therapy selection, especially for those at high NODAT risk post-transplantation. Close monitoring post-transplantation is important, particularly for patients with impaired glucose metabolism and those at risk for NODAT [62].

#### 4.1.2. Antidiabetic Medications

The use of different medications to manage NODAT involves multiple approaches, each with distinct effects and considerations. Insulin therapy in the early months of post-transplantation aims to counter stress hyperglycemia and preserve β-cell function. Studies suggest early insulin supplementation could potentially reduce NODAT cases by safeguarding β-cells [8]. However, larger trials regarding aggressive blood sugar control using insulin in the early transplant phase showed inconclusive outcomes. For mild hyperglycemia after transplantation, antihyperglycemic agents are preferred over insulin [63].

Metformin is recommended as a first-line oral therapy if certain conditions are met for kidney transplant recipients. Its potential benefits, including glucose reduction and improved insulin sensitivity, have been noted, although its safety post-transplantation remains uncertain [64]. Studies exploring metformin’s safety in kidney transplant recipients indicate no significant negative impact on patient or graft survival, and it can be used in subjects with eGFR > 30 mL/min. Yet, the safety and efficacy of metformin post-transplantation require further substantiation through large randomized controlled trials [65]. 

Dipeptidyl peptidase 4 inhibitors (DPP4i) have shown promise in NODAT treatment by potentially improving insulin sensitivity and protecting β-cell mass. DPP-4 inhibitors work by blocking the action of DPP-4, an enzyme that destroys the hormone incretin. Incretins help the body produce more insulin only when it is needed and reduce the amount of glucose being produced by the liver when it is not needed. These inhibitors, like sitagliptin, have demonstrated increased insulin sensitivity in patients with NODAT. Their tolerability and minimal side effects make them a more frequent choice for NODAT treatment, particularly due to their limited interaction with common immunosuppressive drugs [66,67,68]. In a study with a total of 61 patients, they found that initiating sitagliptin promptly in the initial week post-transplant for hyperglycemia (blood glucose > 200 mg/dL) and discontinuing it by the third month in non-diabetic patients led to a notable 18.06% absolute risk reduction in abnormal OGTT outcomes [69].

Glucagon-like peptide-1 receptor agonists (GLP-1 RA) have proven beneficial in improving insulin secretion and reducing hypoglycemia episodes [34]. They offer weight loss benefits and potentially enhance cardiovascular health. However, their use may lead to gastrointestinal issues and requires one to consider their interaction with immunosuppressive drugs [70,71]. GLP-1 RA primarily functions by inhibiting post-meal glucagon secretion and reducing hepatic glucose production while also slowing gastric emptying and suppressing appetite. Notably, in KTRs, these agents offer benefits, including a 1.5–2% decrease in HbA1c, reduced insulin needs, lower postprandial and fasting blood sugars, and a 2 kg weight loss within three weeks [72].

Sodium–glucose cotransporter 2 inhibitors (SGLT2) reduce blood sugar levels and have demonstrated cardiovascular benefits in non-transplant populations. However, their effects and safety in renal transplant recipients remain unclear. While they can effectively control glucose and body weight, especially in patients with good graft function, their potential complications, including urinary tract infections and risks of hypotension or acute kidney injury, warrant careful evaluation [73]. The risk–benefit ratio of using SGLT2 inhibitors in the immediate post-transplant period needs cautious assessment, considering their potential complications, especially in cases of insulin deficiency or acute illness [74]. SGLT2 inhibitors, particularly empagliflozin, have been shown to exhibit positive effects on blood glucose levels and have the potential to slow chronic kidney disease progression. It has also been shown to be useful in KTRs. In the in vitro model, empagliflozin demonstrated renal protection against the adverse effects of tacrolimus, mitigating kidney damage in laboratory settings. Tacrolimus increases SGLT-2 levels, but empagliflozin counteracts this effect by reducing these levels, promoting sugar excretion in urine, enhancing blood sugar regulation, increasing insulin production, and protecting against tacrolimus-induced kidney damage [75,76].

In summary, various medications show promise in managing NODAT post-transplantation, but larger randomized controlled trials are essential to confirm their efficacy and safety in this specific patient population. The choice of medication needs to consider its potential benefits and its interactions with immunosuppressive drugs and the individual patient’s condition to optimize outcomes and minimize adverse effects.

### 4.2. Potential Novel Therapies or Approaches

Novel therapies, extensively studied in the context of T2DM, hold considerable promise for addressing the challenges of managing NODAT. By leveraging advanced insulin formulations, medications targeting compromised mitochondrial function like Imeglimin, and drugs that enhance GLP-1 production, along with oral GLP-1 receptor agonists available in small molecular form, individuals with NODAT can potentially benefit from improved blood sugar control and weight management. Moreover, exploring single-molecule peptides interacting with multiple gut hormone receptors, particularly GIP and GLP-1 co-agonists and potential agonists for GLP-1 and peptide YY, presents opportunities for addressing metabolic challenges in NODAT patients. Thus, the translational potential of these therapies from T2DM to NODAT offers hope for more effective management strategies in this patient population [77].

### 4.3. Lifestyle Interventions and Dietary Considerations

Adopting habits to encourage fat and energy expenditure, engaging in moderate physical activity, and aiming for moderate weight loss are recommended strategies for lowering the risk of type 2 diabetes mellitus [78,79]. In the DIADEM-I study conducted by Taheri et al. in primary care and community settings, intensive lifestyle interventions resulted in significant weight loss within 12 months, diabetes remission in over 60% of participants, and the normalization of blood sugar levels in more than 30% [80]. However, there is insufficient clear evidence regarding the impact of lifestyle changes on the risk of NODAT. Another trial involving 103 kidney transplant recipients, focusing on glycemic control strategies and led by renal dietitians, showed no improvement in surrogate markers of glucose metabolism despite active lifestyle intervention [81]. Nevertheless, recent smaller studies have indicated promising results from moderate-to-vigorous physical exercise and adherence to Mediterranean-style or plant-based diets in potentially reducing NODAT risk [82,83]. 

Boosting physical activity and muscle mass can aid in preventing NODAT. Physical activity stimulates 5-alpha-AMPKs, which improves glucose absorption and fatty acid oxidation and enhances muscle glucose uptake [84]. Lastly, bariatric surgery, such as gastric sleeve, provides considerable advantages for obese patients post-kidney transplantation, offering significant and enduring weight loss along with improvements in comorbidities and graft function. This procedure is favored over the Roux-en-Y method due to its association with lower risks of oxalate stones and decreased absorption of immunosuppressants [85].

## 5. Complications and Long-Term Consequences

### 5.1. Short-Term Complications of NODAT

Studies have indicated that NODAT correlates with diminished inner neurosensory retinal layers, while vascular density remains relatively unchanged. This implies early neuroretinal degeneration might precede vascular alterations due to NODAT [86]. After the onset of NODAT, nephropathy has been detected in transplanted kidneys [87,88]. Additionally, neuropathy is linked to this condition [89].

### 5.2. Long-Term Consequences on Graft and Patient Survival

NODAT significantly impacts patient survival, graft rejection, graft loss, and infectious complications post-kidney transplant. A study involving 173 renal transplant recipients showed that 1-year patient survival rates were notably lower in those with NODAT compared to those without (83% vs. 98%, respectively; *p* < 0.01) [87]. Data from over 11,000 Medicare beneficiaries revealed that NODAT increased the risk of graft failure by 63%, death-censored graft failure by 46%, and mortality by 87% compared to individuals without diabetes [7].

In a retrospective study, they found that patients who started with higher initial insulin resistance (IR) and had an impaired glucose regulation (IGR) of ≥0.092 experienced notably poorer early kidney graft function. Furthermore, it was revealed that IGR served as a predictive indicator for unfavorable kidney graft function one year post-transplant [88].

### 5.3. Impact on Cardiovascular Health

NODAT is associated with a 60% increase in post-transplantation myocardial infarction, an increased risk of cerebrovascular accidents, as well as aortic or lower extremity arterial disease [89,90]. There has been a strong noted correlation between fasting hyperglycemia and cardiovascular (CV) risk post-transplant, akin to associations observed in the general population. The findings imply a continuous relationship between elevated fasting plasma glucose levels and CV risk in transplant recipients, suggesting a notably brief interval between the onset of hyperglycemia, heightened CV risk, and mortality, possibly due to pre-existing cardiovascular disease in patients with post-transplant diabetes mellitus [91]. 

**Figure 2:**
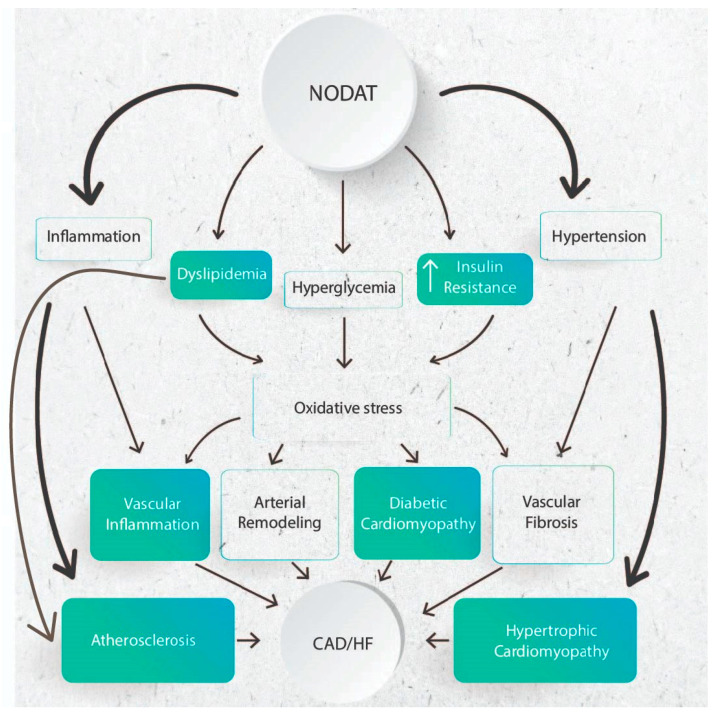
This figure illustrates the cascading effects of NODAT on various physiological processes, particularly hypertension, inflammation, and oxidative stress, ultimately leading to cardiac complications. NODAT is state of hyperglycemia caused by β-Cell dysfunction and increaseed insulin resistance, leads to elevated blood pressure and systemic inflammation. These interconnected factors heighten oxidative stress levels, exerting detrimental effects on cardiac function and health.

NODAT, characterized by hyperglycemia, shares similarities with DM2’s impact on the cardiovascular system. Oxidative stress emerges as a critical factor contributing to the complications associated with NODAT, affecting both microvascular and cardiovascular health. The experimental evidence reviewed here underscores how the metabolic irregularities characteristic of diabetes contribute to an excess production of mitochondrial superoxide. This heightened production serves as a central mediator in the tissue damage seen in diabetes, activating five pathways implicated in complication pathogenesis and directly impairing two antiatherosclerotic enzymes, endothelial nitric oxide synthase and prostacyclin synthase [92] (Figure 2).

Furthermore, the interplay between hyperglycemia and hypertension, often attributed to factors like obesity, insulin resistance, and hyperinsulinemia, underscores a complex relationship. Hyperglycemia itself can instigate changes in vascular function and structure, leading to hypertension. This hypertensive state, in turn, can precipitate conditions such as hypertrophic cardiomyopathy and coronary artery disease (CAD). These insights emphasize the multifaceted nature of cardiovascular complications associated with NODAT and underscore the importance of addressing both hyperglycemia and hypertension in mitigating its impact on cardiovascular health [93].

## 6. Prevention Strategies

### 6.1. Strategies for Preventing NODAT Pre-Transplant

The initial step in averting NODAT involves recognizing the risk factors preceding transplantation. Individuals awaiting kidney transplants necessitate a comprehensive assessment of their diabetes susceptibility before being enlisted. High-risk patients should be advised on preventive measures such as weight management, lifestyle adjustments, monitoring caloric intake, and engaging in physical activity. For individuals at heightened risk of NODAT, like those aged over 45 years [16], individuals with a familial history of diabetes, individuals with metabolic syndrome coupled with elevated triglyceride levels [94], high blood pressure, impaired fasting glucose [95], or those with HCV infection [96], it is recommended to assess HbA1c levels and conduct OGTTs [8,97].

### 6.2. Early Post-Transplant Interventions to Minimize NODAT Risk

HbA1c levels may not accurately reflect blood glucose in the initial phase following a transplant due to factors such as bleeding, bone marrow suppression, or erythropoietin administration in cases of delayed graft function [98]. Jenssen T et al. proposed maintaining fasting plasma glucose below 7 mmol/L (126 mg/dL) and post-meal glucose levels below 10 mmol/L (180 mg/dL) during the early post-transplant period [99]. It is typically advised to aim for HbA1c levels between 7.0% and 7.5% (53–58 mmol/mol) during this time [100].

## 7. Current Ongoing Research in the Field

In terms of ongoing research, one study [101] presents compelling evidence suggesting that human B-cell dysfunction triggered by tacrolimus and Sirolimus in NODAT patients is reversible and preventable. According to the study, the dysfunction induced by these drugs can be reversed upon the discontinuation of the medication and can be prevented through the coadministration of the GLP-1 agonist Exendin-4 (Ex-4). These findings hold significant clinical relevance. Notably, Ex-4 and tacrolimus target overlapping pathways, with tacrolimus inhibiting calcineurin, while GLP-1 receptor (GLP-1R) signaling in β-cells has been demonstrated to activate calcineurin. Findings suggest that activating calcineurin through the GLP-1R in β-cells can counteract the inhibition induced by tacrolimus, thereby averting insulin secretory deficits. These insights provide valuable guidance for managing B-cell dysfunction in post-transplant DM patients, offering potential avenues for therapeutic intervention and improved patient outcomes. However, further corroborative research and clinical trials are imperative to solidify the reliability of these conclusions and establish concrete preventive measures against the adverse effects of tacrolimus and Sirolimus on β-cell function [101].

Recent advancements in genome sequencing have catalyzed extensive investigation into the role of the gut microbiota in various diseases, notably metabolic disorders such as diabetes. Despite strides in comprehending the intricate relationship between gut bacteria and the host, particularly concerning inflammation and disease progression, establishing a definitive causal link remains a formidable task. Dysbiosis, characterized by gut bacteria imbalance, has been associated with diabetes and its complications, including retinopathy, neuropathy, and cardiac diseases [102]. Studies have revealed associations between antidiabetic medications like metformin and the gut microbiome, shedding light on their antidiabetic mechanisms by modulating inflammation, gut permeability, glucose homeostasis, and short-chain fatty acid (SCFA)-producing bacteria [103]. Conversely, the impact of SGLT2 inhibitors on the gut microbiome remains relatively unexplored, contrasting with the well-documented effects of metformin. While some trials have shown no significant differences in microbiome composition between dapagliflozin and gliclazide, others have observed empagliflozin promoting beneficial bacteria, such as Eubacterium and Faecalibacterium, while reducing potentially harmful ones. The early and consistent alterations in gut microbiome and plasma metabolites following empagliflozin initiation suggest adaptability to biochemical shifts, necessitating longer-term assessments and more frequent monitoring for conclusive insights [104]. While certain bacteria exhibit promises as therapeutic targets, further investigation, particularly through human trials, is imperative to explore the efficacy of interventions like prebiotics, probiotics, and fecal microbiota transfer in managing diabetes and its associated complications, which can be applied to NODAT patients.

## 8. Conclusions

NODAT is a prevalent complication following kidney transplantation, with reported incidences ranging from 10% to 50% within the first few years and up to 20% within the first year alone. The underdiagnosis of NODAT is frequent, partly due to the unreliable hemoglobin levels affecting HbA1c measurements. NODAT significantly increases the risk of graft loss, patient mortality, and diminished quality of life, underscoring the importance of the early identification of risk factors and preventative measures. This study evaluates predisposing factors to NODAT, including older age, visceral obesity, ethnicity, viral infections, and the impact of various immunosuppressive medications. Calcineurin inhibitors and mTOR inhibitors are particularly implicated in β-cell dysfunction.

Considering the treatment of NODAT, options include oral antidiabetic agents such as metformin, GLP-1 Ras, and SGLT-2 inhibitors, alongside lifestyle interventions. Metformin is noteworthy for its potential role in managing T2DM by modulating the gut microbiome. Additionally, empagliflozin, an SGLT-2 inhibitor, has shown renal protective benefits against tacrolimus-induced adverse effects in animal studies. There’s also evidence to suggest that the detrimental effects of CNIs on pancreatic function can be mitigated by discontinuing the medication or through the coadministration of GLP-1 agonist Exendin-4 (Ex-4).

Bariatric surgery, particularly sleeve gastrectomy, presents a viable option for both preventing and managing NODAT in obese patients. As research continues to evolve in this domain, it holds promise for enhancing patient outcomes and quality of life post-kidney transplantation.

## Figures and Tables

**Table 1:** This table provides a comprehensive overview of the diagnostic criteria used to identify NODAT.

Test	Result
Fasting Glucose	≥126 mg/dL (7 mmol/L) on more than one occasion.
Random Glucose	≥200 mg/dL (11.1 mmol/L) with symptoms.
Two-hour glucose after a 75 g OGTT	≥200 mg/dL (11.1 mmol/L).
HbA1c	>6.5%

**Table 2:** This table provides an overview of both modifiable and non-modifiable risk factors associated with NODAT, facilitating risk stratification and targeted interventions to mitigate the development of diabetes mellitus after solid organ transplantation.

Modifiable risk factors	Immunosuppression Rejection episodes Obesity Metabolic syndrome Hepatitis C virus infection
Non-modifiable risk factors	Age Ethnicity Male sex Family history of diabetes

## Data Availability

Not applicable.
